# Peri-operative platelet-rich plasma in arthroscopic femoroacetabular impingement surgery: a randomized controlled trial

**DOI:** 10.1093/jhps/hnab001

**Published:** 2021-07-16

**Authors:** Gen Lin Foo, Joshua Sapong Knudsen, Catherine Jane Bacon, Omer Mei-Dan, Mark Owen McConkey, Matthew John Brick

**Affiliations:** 1 Orthosports North Harbour Ltd., AUT Millenium, 17 Antares Place, Rosedale 0632, New Zealand; 2 New Zealand Orthopaedic Association, Level 12, Ranchhod Tower, 39, The Terrace, Wellington 6011, New Zealand; 3 Orthosports North Harbour Ltd. & School of Nursing, Faculty of Medical and Health Sciences, University of Auckland, New Zealand; 4 Division of Sports Medicine and Hip Preservation, Department of Orthopaedics, University of Colorado School of Medicine, Boulder, Colorado, USA; 5 Division of Arthroscopic, Reconstructive Surgery and Joint Preservation, Department of Orthopaedics, University of British Columbia, Vancouver, BC, Canada; 6 Orthosports North Harbour Ltd., AUT Millenium , 17 Antares Place, Rosedale 0632, New Zealand

## Abstract

This study aimed to determine whether the addition of platelet-rich plasma (PRP) during hip arthroscopy improves functional outcomes in femoroacetabular impingement (FAI) surgery. This was a prospective randomized single-blinded trial of arthroscopic hip patients aged between 16 and 50 years with a diagnosis of FAI conducted at a single centre. Patients with any previous hip surgery and significant osteoarthritic changes (Tonnis grade > 2) were excluded. Before surgery, patients were randomly assigned to receive either a PRP injection or a saline placebo. Efficacy was evaluated at 6 months, 1 year and 2 years post-surgery using patient-reported outcomes. The short version International Hip Outcome Tool (iHOT12) was the primary outcome. Recruited patients (*n* = 113) were aged 36.0 ± 10.5 (mean ± standard deviation) years and 56% male. At baseline, iHOT12 scores of the PRP (mean 43.8 ± 22.4) and placebo groups (mean 45.2 ± 21.5) were similar. At a minimum follow-up of 2 years, both groups had improved iHOT12 scores (PRP: mean 83.6 ± 13.4, control: mean 77.1 ± 23.3), with no significant difference in change between the two groups (*P* = 0.19). There were no significant group differences for the change in Non-Arthritic Hip and Hip Disability and Osteoarthritis Outcome Score—Shortform scores between the two groups (*P* = 0.22 and 0.46, respectively). The present study does not support the peri-operative use of PRP in arthroscopic surgery for FAI for mid-term improvement. There were no significant differences in outcome between PRP and placebo groups at 2-year minimum follow-up after surgery.

## INTRODUCTION

Platelet-rich plasma (PRP) is widely used in musculoskeletal conditions and orthopaedic surgery ranging from clinic-based injections for tendinopathies to intra-operative application to augment soft-tissue healing [1, 2]. It has been used in knee osteoarthritis, rotator cuff tears, elbow epicondylitis, anterior cruciate ligament reconstruction, shoulder impingement syndrome and various tendinopathies to date, with an expanding list of indications [3–5]. While available evidence supports the use of PRP in the management of lateral epicondylitis and knee osteoarthritis, there is limited clinically significant evidence for other indications [6, 7]. 

PRP is generated from centrifugation of whole blood to yield a higher than usual concentration of platelets in a small volume of plasma. Platelets contain a range of growth factors and cytokines such as transforming growth factor-β, insulin-like growth factor, platelet-derived growth factor and vascular endothelial growth factor. These are involved in the regulation of chemotaxis, angiogenesis, cell proliferation and differentiation. They have been shown to enhance healing by stimulating angiogenesis and production of collagen [8, 9]. Thus, PRP potentially improves clinical outcomes by improving inflammatory symptoms as well augmenting labral and cartilage healing [10]. However, the use of PRP is still an evolving field and there is a need for more high-quality literature on its efficacy in clinical practice.

The treatment of femoroacetabular impingement (FAI) has made great strides since the introduction of surgical hip dislocation by Ganz *et al.* [11, 12]. Patients with FAI who have failed conservative management are increasingly treated with hip arthroscopy to repair the injured labrum and cartilage as well as the necessary resection of the pincer or cam morphology.

There is a small but expanding pool of studies investigating the use of PRP in hip arthroscopy [13–15]. However, there is limited evidence available regarding the effect of PRP on hip arthroscopy mid-term outcomes (2 years and beyond). Therefore, the purpose of this study is to determine whether the addition of PRP during hip arthroscopy improves functional outcomes in arthroscopic FAI surgery. We hypothesize that the use of PRP in patients undergoing hip arthroscopy for FAI will result in improved clinical outcomes.

## MATERIALS AND METHODS 

This was a prospective randomized single-blinded trial conducted at a single medical centre. Ethics approval was determined by the local institutional review board and informed consent was obtained from all study participants.

The recruitment period was between September 2011 and December 2013 during which the surgeon completed 449 hip arthroscopies. One hundred and thirteen patients with FAI were recruited. Inclusion criteria for this study were ages between 16 and 50 at time of surgery, healthy without any significant systemic disease and with the diagnosis of FAI (which included labrum and cartilage injuries). Exclusion criteria were patients with any previous hip surgery, significant osteoarthritic changes (Tonnis grade > 2) and hip dysplasia (lateral centre edge angle <20°).

Prior to the surgery, patients were randomized to receive either a PRP or a saline placebo. The randomization process utilized computer-generated random numbers, which were then placed in sealed envelopes. Patients were unaware of which group they were in throughout the study. The primary surgeon was also blinded to which group the patients were allocated to. Peripheral blood was taken from both the PRP and placebo group.

Under general anaesthesia, the surgery was performed with the patient in lateral position with a McCarthy hip distractor. Two portals, mid-trochanteric and anterior were used with the occasional distal anterolateral accessory portal for optimal placement of anchors. Labral repair and cartilage procedures (debridement or microfracture depending on the extent of cartilage injury) were performed. Adequate resection of FAI (cam, pincer or mixed) was confirmed on fluoroscopy. Capsular closure was performed for all the patients. A Manovac^®^ drain was left *in situ* at the conclusion of surgery. The drain was placed under direct arthroscopic vision through the anterior portal and secured with the portal nylon stitch and adhesive dressing.

The PRP was prepared using the Plasma Rich in Growth Factors (PRGF)-Endoret system V (BTI, Spain). The withdrawn peripheral blood underwent 8 min of centrifugation followed by separation of the plasma fraction. A single use PRGF pipette was utilized to separate the layers and the superficial platelet poor plasma was discarded. The platelet-rich component was placed in a plain tube. The remaining buffy coat and red cells were discarded. The PRP component was stored in a fridge overnight within temperature ranges of 2–8°C.

The next day, the patients received either a PRP or saline injection via their hip Manovac^®^ drain depending on their allocated group. The drain was secured with adhesive dressing and patient activity within the first 24 hours after surgery was kept minimal to reduce the risk of the drain dislodging. The PRP was activated with calcium chloride (10% injection 1 g in 10 ml; Phebra, NSW, Australia) at a ratio of 50 μg (0.05 ml) per 1 ml of PRP prior to injection. The drain was flushed with normal saline before and after the PRP injection, with removal immediately after. The patients were discharged the day after their surgery. Partial weight bearing with crutches was prescribed for 2 weeks with an additional 4 weeks for patients who had underwent microfracture. Immediate range of motion exercises was encouraged.

Patients were then followed up in clinic at the 1-week, 3-month and 1-year post-surgery time points. Performance score questionnaires, including the iHOT12, NAH (Non-Arthritic Hip Score), HOOS-SF (Hip Disability and Osteoarthritis Outcome Score—Shortform) were sent to patients at the pre-operative, 6 months, 1 year and 2 years post-operative time points. As part of their routine follow-up, patients also had these questionnaires sent out to them annually after their surgery.

Patient satisfaction was also assessed by an additional question on their likelihood of having the surgery again on their contralateral hip if symptomatic. The range of responses was ‘definitely yes, probably yes, possibly not and definitely not’. Any conversions to total hip arthroplasty (THA) were also noted. 

Patient-reported outcomes were collected with the use of an online scoring system, Socrates (Ortholink Pty Ltd, NSW, Australia). Patients who opted for traditional mail options were accommodated for.

The primary outcome measure was the change in iHOT12 performance scores in the PRP group compared to the placebo group [16]. Further outcome measures include the NAH and HOOS-SF as well as any revision surgeries including conversion to total hip replacement [17, 18].

A minimum sample size of 51 per group was calculated using G*Power 3.1 (Dusseldorf, Germany) to detect an effect size of 0.5. This is equivalent to a between-group difference in the change of iHOT12 (Short version International Hip Outcome Tool) of 12.5 points, based on a standard deviation (SD) of the change of 25 points, a statistical power of >80% and an alpha error rate of <0.05 [19, 20].

Changes in outcome scores from baseline to 2 years minimum follow-up were checked for violation of assumptions of normality by visual inspection of their distribution and analysis of skewness and kurtosis *z*-scores for those falling outside 9% confidence limits. We used a two-way Analysis of variance (ANOVA) to compare changes in scores between the PRP and placebo groups from baseline to 2 years minimum follow-up. *P*-value <0.05 was considered significant. Statistical analyses were performed with IBM SPSS Statistics for Windows, version 26 (IBM Corp., Armonk, NY, USA).

## RESULTS

**Fig. 1.**
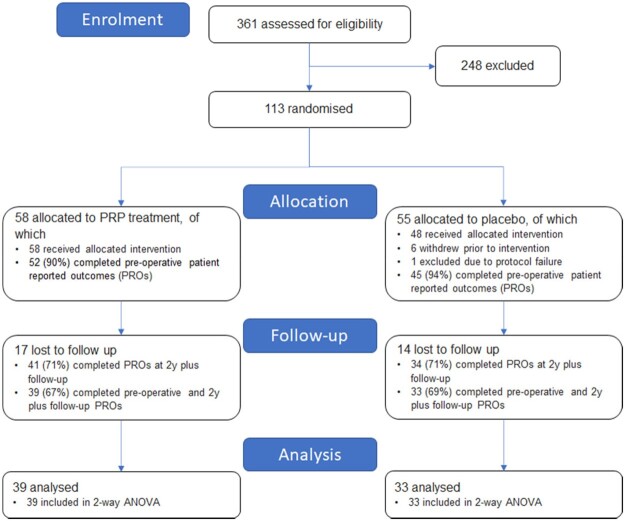
Flow of participants in study.

From a pool of 361 eligible procedures, 113 patients were enrolled and randomized (Fig. 1). Of those, 58 patients were allocated to the PRP treatment and 55 were allocated to placebo. Of the placebo group, one patient was excluded from the study due to protocol failure (drain was pulled out prior to injection) and six withdrew from the study after randomization but before intervention.

Baseline characteristics were well matched between the randomized groups. The demographics of each group at baseline are summarized in Table I.

**Table I.** Baseline demographic and clinical characteristics of patients randomized to PRP and placebo groups

Characteristics	PRP (*n* = 58)	Placebo (*n* = 48)
Gender (female)	26 (44.8)	21 (43.8)
Surgery side (left)	25 (43.1)	25 (52.1)
Age (years)	36.1 (±10.5)	35.9 (±10.5)
Body mass index^a^ (kg/m^2^)	23.2 (±7.78)	24.3 (±5.50)
Lateral centre edge angle (degrees)	32.8 (±5.5)	33.8 (±6.2)
Labral pathology		
None/normal	11 (19.0)	5 (10.4)
Hypoplastic/hyperplastic	8 (13.8)	2 (4.2)
Degenerative/calcified	8 (13.8)	9 (18.8)
Partial tear	22 (37.9)	25 (52.1)
Full thickness tear	9 (15.5)	7 (14.6)
Tonnis score (Grade 1 only)	11 (19.0)	8 (16.7)
Acetabular chondral lesion		
None/normal	5 (8.6)	4 (8.3)
Early delamination	27 (46.6)	22 (45.8)
Full thickness (<1/3 rim to fossa)	17 (29.3)	13 (27.1)
Full thickness (1/3–2/3 rim to fossa)	9 (15.5)	9 (18.8)
Crossover sign	16 (27.6)	12 (25.0)

Data are mean (SD) for continuous variables and frequency (%) for categorical variables.

^a^ BMI data available for 37 of PRP group and 30 of placebo group.

**Fig. 2.**
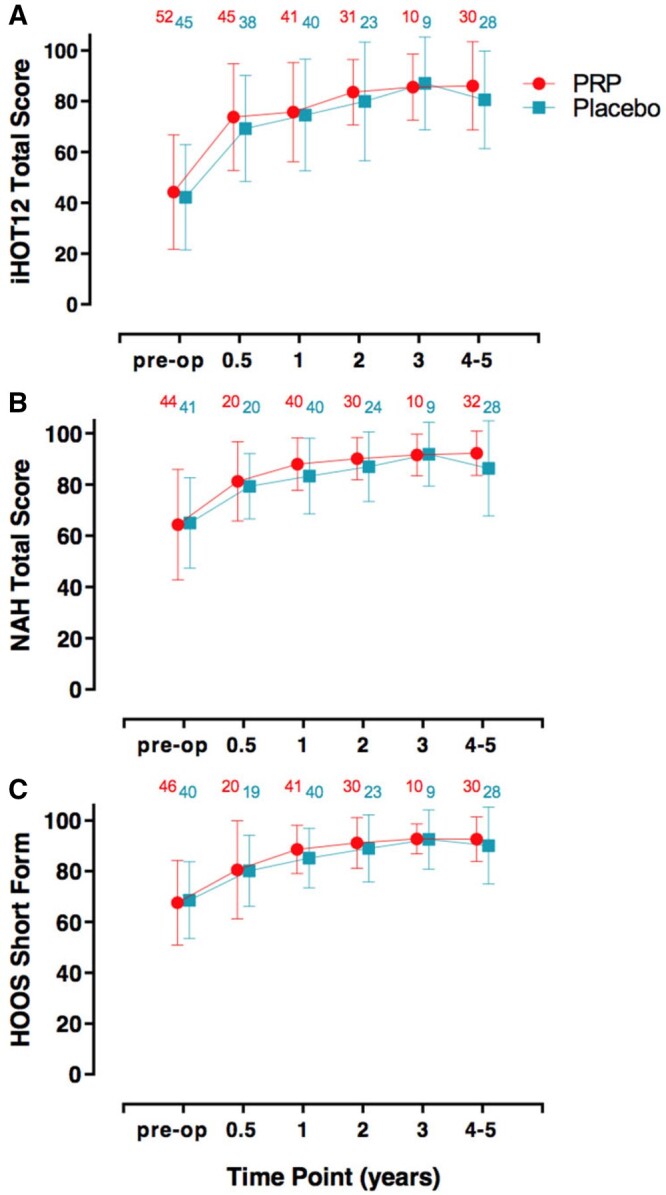
Patient-reported outcome measures at pre-operation, 6 months, 1 year, 2 years, 3 years and 4–5 years. A: IHOT-12 score, B: NAH score, C: HOOS-SF score. Numbers above data points indicate the sample size available from the 58 in the PRP group and 48 in the place group. Error bars are 95% confidence intervals.

There were no significant differences between the two groups at the different time points 6 months, 1 year, 2 years, 3 years and 4–5 years after surgery. This is illustrated in Fig. 2A–C.

A ‘2-year minimum follow-up’ variable was computed by including the earliest available performance scores between 2 and 5 years (inclusive) post-surgery. The PRP group reported an iHOT12 mean of 83.6 (SD = 13.4, *n* = 39) and the placebo group reported an iHOT12 mean of 77.1 (SD = 23.3, *n* = 33) for this variable. There was no significant difference in change between the two groups at 2 years minimum follow-up (*P* = 0.19).

The other performance measures (NAH and HOOS-SF) also showed no evidence of difference between the PRP and placebo groups (Table II).

**Table II.** Outcome measures pre-operatively and 2-year minimum follow-up

		PRP			Placebo		Difference in change	*P*-value^a^
	*n* (%)	Pre-op	2-year min	*n* (%)	Pre-op	2-year min	Mean (95% CI)	
iHOT12	39 (67)	43.8 ± 22.4	83.6 ± 13.4	33 (69)	45.2 ± 21.5	77.1 ± 23.3	7.97 (−4.06 to 20.0)	0.19
NAH	33 (57)	66.6 ± 19.6	90.8 ± 8.4	30 (63)	68.2 ± 17.3	86.6 ± 17.9	5.82 (−3.38 to 15.0)	0.22
HOOS-SF	34 (59)	68.7 ± 15.9	91.0 ± 9.9	29 (60)	69.8 ± 15.5	88.5 ± 16.7	3.55 (−6.08 to 13.2)	0.46

Data are sample size (percentage of group); mean ± SD and 95% confidence interval for difference in change in scores between PRP and placebo groups. Data are only for patients with both scores available, sample sizes shown. 2-year min is follow-up at 2 years if available, otherwise in order of priority at 3 years, or 4–5 years.

^a^ *P*-value is for pre-operative to follow-up change by group interaction.

Patient satisfaction assessment showed that the majority of patients in each group would have had the operation again with 87.8% and 89.4% in the PRP and placebo group, respectively, answering ‘definitely and probably yes’ (Table III). There was no significant difference in the response between the two groups (*P* = 0.55).

**Table III.** Patient satisfaction—would you have operation again?

	PRP	Placebo
Definitely yes	26 (63.4%)	18 (52.9%)
Probably yes	10 (24.4%)	9 (26.5%)
Possibly not	5 (12.2%)	7 (20.6%)
Definitely not	0	0
Total response	41 (70.7%)	34 (70.8%)

Percentages are of those in each group who responded to the item except for total response which is of all participants.

A total of 11 patients had revision surgery, all within 5 years of their index surgery. Two out of the seven in the PRP group and two of the four in the placebo group had their revision surgery within 2 years. There was no significant difference in conversion to THA between the two groups (Table IV).

**Table IV.** Subsequent ipsilateral hip surgery

	PRP	Placebo	*P*-value
Follow-up duration (years)	7.41 (±0.61)	7.49 (±0.62)	0.51
Revision/reoperation	6	3	0.45
Within 2 years	2	1	0.67
Duration to revision/reoperation (years)	2.67 (±0.76)	2.67 (±1.76)	1.00
Conversion to total hip replacement	1	1	0.89
Within 2 years	0	1	0.27
Duration to total hip replacement (years)	3.45	1.24	—

Data are numbers of operations and mean (±SD) for durations.

## DISCUSSION

Our blinded, randomized controlled trial showed no evidence that PRP injections given the day after hip arthroscopy for FAI surgery improved mid-term patient outcomes. Patient-reported outcomes at 6 months, 1 year, 2 years and 2-year minimum follow-up did not differ significantly between PRP and placebo groups.

The PRP that we used was not tested in each case. However, previous studies have reported that the BTI PRGF-Endoret system to be leucocyte-poor and approximately 2 to 3 times the baseline platelet concentration [21–23]. Reviews of concentration yields from different PRP preparation systems have found that the final product concentrations can vary widely between systems, with over 40 different commercial systems available [24–26]. In addition, no universally accepted system of PRP classification exists [27]. As the process of PRP preparation affects its final contents and thus its therapeutic potential, we recognize that different preparation methods could lead to differing results.

Mannava *et al.* [28] describe a different protocol for preparing and injecting active PRP for hip arthroscopies. They also administer the PRP immediately after completion of hip arthroscopy, noting that there is a risk that PRP is diluted or washed away due to arthroscopic fluid in the joint. We designed our study to have the PRP administered the day after surgery to mitigate this issue. To date, there is no consensus on the timing of PRP administration to reduce the risk of dilution from arthroscopic fluid. The risk is highest with intra-operative administration and diminishes with time as the fluid resorbs and capsule heals. Administration of PRP in the clinic 1–2 weeks after surgery when the joint is dry eliminates the dilution and oozing out factors but comes with the downside of additional workflow and costs in the outpatient setting.

Thus far there have been limited long-term studies on the effect of PRP as an adjunct in hip arthroscopies for FAI, with most indicating no significant difference in longer-term outcomes. Redmond *et al.*’s [13] prospective comparative study of intra-operative PRP versus bupivacaine injection on patients undergoing hip arthroscopy for labral tears concluded that PRP administration did not improve patient outcomes 2 years after surgery. Patients in the PRP group had lower modified Harris Hip Score (mHHS) and worse pain scores at 2 years post-operatively. There was no significant difference between study and control groups for conversion to total hip replacement or revision.

Results from studies on shorter-term outcomes are mixed. Rafols *et al.* [14] reported significantly lower post-operative pain scores 48 hours post-surgery and fewer joint effusions 6 months post-surgery. However, there was no significant difference in labral integration at 6 months. In a different study, Giordano and Snibbe [29] noted that the HOOS activities of daily living subscale (HOOS-ADL) was significantly higher in patients treated with PRP at 1 month.

In a randomized double-blind study, Mardones *et al.* [30] found no significant differences in pain or oedema in PRP versus control groups at 7 and 14 days post-surgery. This finding was corroborated by LaFrance *et al.* [15] who saw no significant difference in thigh diameter 1-week post-surgery in the PRP versus saline control groups. In the same study, there were also no significant differences in patient-reported outcomes for NAH, mHHS and HOOS at 1, 3, 6 months and 1 year post-surgery.

Some studies note a significantly lower presence of ecchymosis in PRP groups compared to control 7 days after single hip arthroscopies [15, 29, 30].

### Strengths

The strengths of this study include the large number of patients undergoing surgery and the single-blinded, randomized nature of the study. To the best of our knowledge, this is the largest study to assess the longer-term effects of PRP injections as an adjunct in hip arthroscopy in a single-surgeon, prospective, blinded, randomized and controlled setting.

All the patients underwent surgery by an experienced senior surgeon with a standardized technique and post-operative rehabilitation protocol. This reduces any confounding factor from variation in surgical technique and experience. The same PRP preparation and method of administration was also applied to all participants.

We also used additional outcome measures on top of our primary outcome measure, iHOT12. The NAH and HOOS-SF results were similar to iHOT12 which provides additional support to our findings that PRP did not alter the outcome of FAI hip arthroscopy.

### Limitations

Our study has several limitations. The first is the lack of complete follow-up. Only 54 of the 105 patients had complete data at 2 years post-surgery. Thus, we computed a constructed variable, the 2 years minimum follow-up with the aim of getting an improved longer-term measure. This attrition led to a reduced power to detect a statistically significant difference in the patient outcomes at 2 years exactly.

Other limitations include lack of post-operative imaging to assess healing between the two groups.

The other limitation is the use of only one method of PRP preparation without quantifying the concentration of platelets delivered. Unfortunately, this is a limitation of many PRP studies, and furthermore, there is no universally accepted standard for PRP preparation.

We used a drain to administer the PRP the day after the surgery. There is a theoretical risk that the drain could have dislodged from the capsule overnight. However, the risk is minimal as the drain was secured with adhesive dressing and patient had minimal activity in the first 24 hours post-operatively. Patients were not put on continuous passive motion or stationary bicycle within this period. We also perform a routine capsule closure and recognize that despite this, the injected PRP can still leak out.

## CONCLUSION

In conclusion, we found no significant differences in outcome between PRP and placebo groups at 2 years plus follow-up after surgery. The present study does not support the peri-operative use of PRP in arthroscopic surgery for FAI for mid-term improvement.

## ACKNOWLEDGEMENTS

The authors would like to acknowledge the assistance of Abbey C. Lissaman in the acquisition of patient-reported outcome data.

## CONFLICT OF INTEREST STATEMENT

None declared. 
